# Tracking Existing Factors Directly Affecting the Reproduction of Bumblebees: Current Knowledge

**DOI:** 10.3390/insects15090654

**Published:** 2024-08-30

**Authors:** Xiaomeng Zhao, Jingxin Jiang, Zilin Pang, Weihua Ma, Yusuo Jiang, Yanfang Fu, Yanjie Liu

**Affiliations:** 1 College of Animal Sciences, Shanxi Agricultural University, Taigu, Jinzhong 030801, China; zhaoxm1027@sxau.edu.cn (X.Z.); 18294245569@163.com (J.J.); 18234685297@163.com (Z.P.); jiangys-001@163.com (Y.J.); 2 College of Horticulture, Shanxi Agricultural University, Taiyuan 030031, China; mawh1997@163.com; 3 HeBei Provincial Animal Husbandry Station, Shijiazhuang 050035, China; fyf781018@163.com

**Keywords:** wild bumblebee, reproduction, colony development, breeding

## Abstract

**Simple Summary:**

Bumblebees are important pollinators of both natural and agricultural ecosystems. The quality of their colonies is highly dependent on the reproductive success of the queens and males. This process can be influenced by numerous factors, both positive and negative, some of which directly affect reproductive processes. In this paper, we review current studies on environmental and biological factors that directly affect bumblebee reproduction, including floral resources, pathogens, pesticides, worker behavior, species competition, and hormonal and genetic influences. More studies, particularly those focusing on bumblebees themselves, are needed to understand the effects of these factors and other potential elements. This understanding is essential in order to meet the demands of agricultural pollination and address the decline in wild bee pollinators worldwide.

**Abstract:**

Bumblebees are primary social insects and a vital class of pollinating insects. Their distinctive reproductive mode is characterized by the independent initiation and construction of the nest by the queen and the subsequent production of sufficient workers, males, and gynes following colony development. After successful mating, the queen transitions to the first phase of its annual life cycle. The reproductive processes are directly influenced by environmental factors, including floral resources and pesticides. Moreover, the reproductive level is regulated by biological factors, particularly the role of workers, who participate in egg laying and pass on their genetic material to the next generation of queens. Successful reproduction can only be achieved by maintaining colony development under natural or artificial breeding conditions. Consequently, understanding the known factors that influence bumblebee reproduction is essential for developing conservation strategies for wild bumblebees and for successfully breeding diverse bumblebee species. Breeding various bumblebee species is crucial for in-depth research into known factors and for further exploration of other potential factors, which will also help to meet the demand for pollination in agricultural facilities globally.

## 1. Introduction

Bumblebees are one of the most important economic and ecological pollinators and have been studied for several years [1]. A bumblebee colony comprises females differentiated into two castes (queens and workers) and males. Compared to workers, queens (including workers and young queens) are larger in size, live longer, and produce female offspring. However, both groups are capable of producing male offspring [2]. Two crucial points are identified during colony development. The “switch point” is the date of the first haploid egg laid by the queen. The “competition point” is the date of the first haploid egg laid by a worker [3,4,5]. Under the habitat conditions of temperate regions and areas near the poles, young queens mate with sexually mature males, hibernate underground for several months, and build new nests to initiate a new life cycle [6]. Because of their commercial value, lower production costs, higher yields, and improved fruit quality, at least five bumblebee species have been artificially bred [7]. Whether naturally occurring or domesticated, the queens’ reproductive processes involve overwintering and establishing the next generation of queens. These processes are affected by many factors, such as the reproductive ability of queens, pathogens, environment, worker reproduction, males, and competition with introduced bumblebee species. 

**Figure 1:**
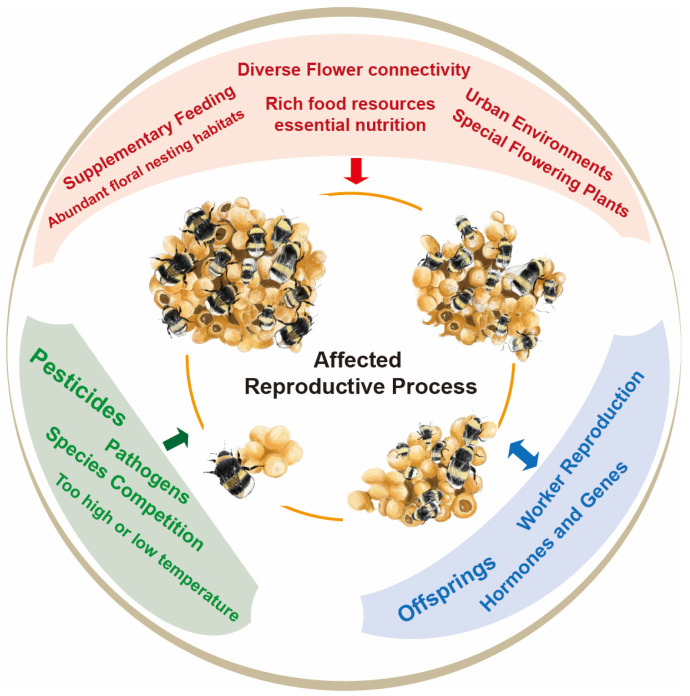
Interaction between direct impact factors and relevant bumblebee reproductive processes. The red arrow denotes a positive effect, the green arrow indicates a negative impact, and the blue arrow represents a positive or negative interaction.

Currently, most relevant studies have focused primarily on *Bombus terrestris*, *B. impatiens*, and *B. lantschouensis* [8]. Here, we review the currently known potential factors, including nesting habitat, flower connectivity, food resources and nutrition, harmful environmental factors, bumblebees themselves, worker reproduction, temperature, competition, and related hormones and genes that directly affect bumblebee reproduction (Figure 1). This review aims to aid the breeding of diverse bumblebee species, advance research on the reproduction of primary social insects, and promote the conservation of wild bumblebee populations.

## 2. Impact of Environmental Factors on Reproduction of Bumblebee

### 2.1. Nesting Habitat and Flower Connectivity

In wild bumblebees, flower abundance determines the reproductive switch point in colonies inhabiting meadows [9]. Diverse landscapes and pollen diets facilitate bumblebee reproduction, including the production of numerous new queens [10]. Conversely, agricultural intensification, loss, and fragmentation of natural and semi-natural habitats are believed to negatively affect bumblebee populations, and a shortage of flower resources in less complex landscapes may negatively affect bumblebee reproduction [11]. The flower resources of simple landscapes can support colony establishment and initial growth of bumblebees. However, the simplification or abandonment of complex landscapes may also threaten bumblebee populations in neighboring simple landscapes [12]. Larger fields of organic agriculture with abundant flower resources benefit *B. terrestris* reproduction and colony growth [13]. The available floral resources near *B. terrestris* colonies positively affect the production of workers and males [14]. In addition, late- and mass-flowering plants in nature or flower strips can promote the production of bumblebee males and gynes, which may overcome poor resources in the late season [15]. Furthermore, bumblebee colonies in urban environments with gardens produce more males and gynes than those in agricultural areas [16], which may result in higher bumblebee nest densities [17,18]. The positive effects of urban environments on bumblebees also depend on the available floral resources [19]. Hence, diverse flower resources greatly facilitate bumblebee colony development regardless of the habitat type. Urbanization is thought to protect bumblebees from the adverse effects of intensive agricultural development.

Several studies have shown that some special flowering plants, such as oilseed rape, may play a positive role in the reproduction of bumblebees. Early mass-flowering oilseed rape had a positive effect on colony growth but did not influence the sexual offspring of *B. terrestris* colonies [20]. Intensive oilseed rape cultivation was more beneficial to the reproductive output of *B. terrestris* compared to semi-natural old apple orchards in southeast Latvia near Krāslava [21]. Additionally, *B. impatiens* colonies in the Phacelia landscapes grew faster, gained more mass, and produced more gynes [22]. Mass-flowering sunflowers increase the number of *B. impatiens* colonies [23]. Furthermore, some studies have shown that artificial cultivation of landscape plants, especially flowers, is beneficial for bumblebee reproduction and colony development [24,25]. Local floral dominance is more critical than flower richness for bumblebee reproduction and colony growth of bumblebees in grasslands [26]. Therefore, agricultural landscapes with dominant or specific mass-flowering plants might benefit bumblebee reproduction; however, this beneficial effect may be short-lived, ending once the flowers wilt.

### 2.2. Food Resources and Nutrition

Artificial supplementary feeding studies have demonstrated that diverse foods have positive effects on bumblebee reproduction. Supplementary feeding with nectar and pollen can increase the number of gynes to twice that in wild bumblebee colonies [27]. Under semi-natural conditions, supplementary feeding of nectar and pollen can increase the numbers of males and daughter queens in *B. terrestris* colonies [28]. In addition, pollen and nectar supplementation throughout all stages of the *B. terrestris* colony has reportedly resulted in large numbers of workers, males, and gynes [29]. *B. terrestris* colonies exhibit the highest growth rates and have large numbers of males and females in fields near flower strips compared to those located farther away [30]. In addition, artificial rearing studies have shown that *B. terrestris* colonies fed a mixture of wild apricot, oilseed rape, buckwheat, and sunflower pollen can produce a more significant number of larger offspring than those fed any of the above single-pollen diets [31]. 

Regardless of the richness, abundance, or dominance of flower resources that benefit bumblebee reproduction, the constant protein:lipid ratio (P:L) in pollen collected from various flowers may be the core nutritional factor affecting the reproductive output and colony growth of *B. terrestris* and *B. impatiens*. This is because bumblebee workers collect pollen at a fixed P:L ratio regardless of the diversity of flower resources available [31,32,33,34,35]. To some extent, P:L also shapes the foraging behavior of workers, which is associated with the types of flower resources available [36,37] and broods in the colony [38]. Pollen with high crude protein content and essential amino acids facilitates the oviposition and colony formation of native *B. breviceps* [39]. The essential amino acids also determine the nutritional status of adult *B. terrestris* workers [40]. Higher protein and amino acid concentrations significantly enhance the larval growth rate and result in larger larvae in microcolonies of *B. terrestris* [41,42]. Sunflower pollen with low protein and essential amino acid contents impedes the growth and development of *B. terrestris* colonies [43]. Poor nutrition can result in low-mass larvae and pupae, as well as over-ejection of larvae in *B. terrestris* [44]. The negative effect of poor nutrition on early-stage queens is irreversible and accompanies their entire life cycle [45]. The collective data indicate that habitat and local plant species determine the nutritional intake of local bumblebees, such as *B. terrestris* [46,47]. 

### 2.3. Pesticides

In addition to poor flower resources, pesticides also have a significant negative effect on bumblebee reproduction. Diverse pesticides, especially neonicotinoids, have been overused following the development of agriculture, which severely threatens pollinators, including bumblebees. An investigation of a European landscape revealed that agricultural pesticide residues in pollen resulted in lighter bumblebee colonies with fewer offspring, especially in simplified landscapes [48]. Imidacloprid is a neonicotinoid that impairs fecundity and severely reduces brood production in bumblebee microcolonies [49]. Exposure to field-realistic concentrations of imidacloprid causes bumblebee colonies to grow more slowly and produce fewer offspring, and results in queens spending less time caring for broods [50]. Regardless of whether the imidacloprid exposure was at experimental or field levels, treated *B. terrestris* colonies suffered a serious reduction in colony weight and the number of daughter queens [51]. Furthermore, reportedly, imidacloprid exposure significantly reduced the mating success rate and the competitiveness of *B. terrestris* males during the mating process [52]. 

Another well-known neonicotinoid, thiamethoxam, can severely impair reproduction in queen-right colonies and microcolonies. In one study, maximum field exposure caused delays in colony start-up and resulted in the production of fewer eggs and no larvae [53]. Thiamethoxam exposure also resulted in the death of nearly one-third of queens that laid eggs and initiated colonies [54]. In addition, exposure to thiamethoxam reportedly damaged sperm viability and hypopharyngeal gland development in *B. terrestris* [55]. Exposure to high doses of thiamethoxam can significantly reduce the number of eggs and larva and impair the growth of *B. terrestris* microcolonies [56,57]. Therefore, thiamethoxam exposure affects the reproductive phase of the bumblebee life cycle. These data form the basis for the suggested ban on the application of neonicotinoids such as imidacloprid and thiamethoxam in crop production worldwide. However, thiacloprid poses a low risk to bumblebee reproduction in mass-flowering clover fields [58]. We suspect that sufficient nutrition compensates for the adverse effects of low-dose thiamethoxam on bumblebee reproduction. 

In addition to neonicotinoids, other pesticides can severely damage the reproductive capacity of bumblebees. Field-dose exposure to sulfoxaflor resulted in fewer workers and sexual offspring during the early and late stages of *B. terrestris* colony cycle [59]. Under nutritional stress, sulfoxaflor exposure significantly impaired worker survival, egg laying, and larval production in *B. terrestris* colonies [60]. Sublethal doses of chlorantraniliprole negatively affected male production in *B. terrestris* microcolonies [61]. Moreover, less toxic fungicides negatively affected the number of workers in *B. impatiens* colonies [62]. In addition to affecting offspring production, the negative impact of pesticides on the behavior of foragers and nurses may impair colony development by limiting nutrient delivery [63]. Hence, both insecticides and microbicides are harmful to various aspects of bumblebee reproduction, including colony development, egg production, larval development, sexual offspring, and copulation. Therefore, pesticides should not be used and the flowering phase should be avoided to protect artificial or wild bumblebee pollinators during agricultural production. 

### 2.4. Temperature

According to an analysis of 21 bumblebee taxa, temperature is an important factor that affects individual body size and colony development [64]. Suitable higher temperatures, such as 33 °C, reportedly facilitated the production of daughter queens during artificial breeding of *B. terrestris* [65]. Higher temperatures from 30 to 32 °C, rather than extremely high temperatures from 34 to 36 °C, increased male production in *B. terrestris* microcolonies [66]. Short-term exposure to extremely high temperatures of 45 °C can impair the fertility of male *B. impatiens* [67]. Moreover, the environmental temperature significantly affected mating success [68]. An optimal nest temperature is required to develop diverse broods in bumblebee colonies [6,69]. In addition, under artificial breeding conditions, cold storage ranging from 2 to 4 °C or carbon dioxide (CO_2_) treatment has been used to break diapause and activate the ovaries of the queens [70,71]. The combined use of CO_2_ and cold storage facilitated egg laying at earlier timepoints [72]. Temperature data show that wild and artificially bred bumblebees require an optimum temperature, which varies among species inhabiting different habitats, to support egg laying, nest building, larval development, queen diapause, and other events.

## 3. Impact of Biological Factors on Reproduction of Bumblebees

### 3.1. Species Competition 

Apart from environmental factors, inter-genus and inter-species competition also significantly impede bumblebee colony development. When bumblebee colonies are located near the honeybee apiary, they tend to produce lighter, fewer, and smaller gynes, and the offspring sex ratio becomes more male-biased [73]. Placing honeybee colonies in or near the habitats of *B. pascuorum*, *B. lucorum*, *B. lapidarius*, or *B. terrestris* eventually leads to bumblebee colonies that produce smaller workers [74]. Another study showed that the introduced *Apis mellifera* negatively affected the foraging and male and female production of *B. occidentalis* [75]. In particular, in simplified landscapes with limited food resources, managed honeybees can significantly suppress bumblebee densities [76]. 

The commercial species *B. terrestris* is widely used for the pollination of greenhouse crops worldwide, which has caused competition with some local bumblebee species [77]. A study described that introduced *B. terrestris* can affect foraging behavior and hinder the normal reproduction and colony development of native bumblebee species [78]. Moreover, the known cross-species hybridization between introduced *B. terrestris* and native bumblebee species has also been shown to disturb the reproduction and colony founding of *B. hypocritus*, *B. h. sapporoensis*, *B. ignitus*, and *B. lantschouensis* [79,80,81]. Hence, more work should be conducted to domesticate and breed local bumblebee species rather than introduce commercially alien bumblebees to meet the pollination needs of crops [82].

### 3.2. Different Castes of Bumblebees 

In addition to environmental factors, workers directly affect the reproductive state of the queen. During the early stage of *B. terrestris* colony, the presence of workers accelerates the activation of the ovaries and egg laying by the queen, contributing to nest success [83]. Worker appearance can also regulate the behavior of queens from feeding on larvae to specialized oviposition by altering the expression of the brain genes of *B. terrestris* queens [84]. Larger workers are beneficial for the collection of floral resources and colony development [85]. Males and queens play key roles in the reproductive success of bumblebees. The reproductive status of *B. terrestris* determines the copulation success [86]. Moreover, mating success, sperm quality, sperm transfer, mating behavior, mating plugs, and the immune status of males are all crucial for bumblebee reproduction [69]. Successful mating can induce the upregulation of genes involved in sperm storage in *B. terrestris* queens [87], which is affected by the lengths of the fore and hind tibiae of mature males [88]. Different *B. terrestris* males possess variable sperm lengths that are positively correlated with the body size of male offspring [89]. Furthermore, the mating plug formed by a male hinders copulation of the mating queen with other males, thereby determining sperm diversity and reproductive output of the queen [90]. However, males are capable of copulating more than once, and non-virgin males will contribute to the queens’ success in founding colonies, producing workers and males, compared to queens mated with virgin males [91]. After successful mating, some species that inhabit temperate regions or areas near the poles must undergo a diapause period to initiate the life cycle of a normal colony [6]. Diapause does not affect the weight of the fat body of queens or the number of offspring to a certain extent [92,93,94]. The duration of diapause affects the production of varying quantities of offspring in *B. terrestris* [95]. 

### 3.3. Pathogens

Concerning pathogens, such as parasites, bacteria, and viruses, can also damage the reproduction of bumblebees. The main bumblebee parasite, *Crithidia bombi*, can inhibit ovarian development and oviposition of workers in queen-right colonies of *B. terrestris* [96]. High doses of *C. bombi* may cause a decline in daughter queen production in colonies [97]. *C. bombi* significantly reduces the success rates of queen diapause, colony founding, male production, and colony size [98,99]. Under starvation conditions, *C. bombi* can cause severe mortality in *B. terrestris* workers [100]. The parasite *Nosema Bombi* has a negative effect on the *B. terrestris* queen-building colonies, colony size, longevity of workers and males, and the vitality of daughter queens and males [101,102,103]. *N. bombi* reportedly significantly reduces gyne production and increases male mortality, eventually leading to a smaller colony size of *B. lucorum* [104]. Serious infections caused by parasitic conopid flies can increase mortality in *B. terrestris* and *B. pascuorum* workers [105]. Both *C. bombi* and *N. bombi* infections have been associated with an increased number of *B. terrestris* males [106]. Parasites have been shown to contribute to the decline in native bumblebees in North America [107]. *Apicystis bombi* infestation can significantly threaten the survival of post-diapause *B. pratorum* queens [108]. Moreover, bacteria and fibrous fungi, including *Bacillus* sp., *B. cereus*, *B. fusiformis*, *B. pumilus*, *B. megaterium*, *B. subtilis*, *Paenibacillus glycanolylicus*, and *Ascosphaera* sp., are thought to be harmful to *B. terrestris* larvae [109]. Oral infections with the Kashmir bee virus or Israeli acute paralysis virus have been reported to delay the development of *B. terrestris* microcolonies and offspring production [110]. Although other viruses that appear in honeybee colonies can also infect bumblebees, to date, there is no evidence of an adverse impact on bumblebee reproduction [111,112,113]. Moreover, Varroa destructor Macula-like virus, Lake Sinai virus, and some new RNA viruses have been identified in wild bumblebees and other wild bee species [113,114]. However, we suspect that these pathogens could impede bumblebees’ reproductive capacity. These parasites and viruses may be transmitted from managed honeybees or commercially pollinated bumblebees to wild bumblebees and other bee species through shared flowers and foraging areas [115,116,117,118]. Regardless of whether the bees are managed or wild, they can serve as potential hosts for pathogens, suggesting that an interconnected network of pathogen threats exists within and among bee species [119]. 

### 3.4. Worker Reproduction 

Although direct contact of some non-volatile pheromones and chemical secretions of queens, as well as worker policing, can suppress the male population of reproductive workers [4,120,121,122,123,124,125,126], 5% of males are produced by workers during the competition phase in queen-right *B. terrestris* colonies [127]. Eggs laid by workers possess vitality equal to that of the queen [128]. Furthermore, worker-born males have equal sperm viability and copulation ability to those born from the queen, which contributes to the colony developmental index of the next generation, including queen egg laying and colony foundation [129]. Interestingly, one study revealed that in *B. wilmattae*, colony workers dominate male production rather than the queen [130]. However, in *B. terrestris* colonies, reproductive workers do not influence the production of new queens [131]. The parasitic bumblebee *B. bohemicus* suppresses the reproduction of host workers to ensure reproductive success without a host queen [132]. Therefore, reproductive workers have a non-negligible influence on bumblebee colonies by dominating or expanding male production. We hypothesized that worker reproduction positively affects the queen’s reproductive level and colony development in bumblebees, potentially increasing bumblebee species’ genetic diversity.

## 4. Hormones and Genes 

Both bumblebee queen and worker reproduction are influenced by the levels of juvenile hormone (JH), which serves as the major gonadotropin and plays an essential role in their reproductive processes [133]. JH from the queen can spread to the epicuticle and inhibit the reproduction of dominant workers [125]. JH directly inhibits larval differentiation into gynes in *B. terrestris* colonies [134]. Moreover, JH regulates the brain-reproduction trade-off in bumblebees; dominant workers exhibit naturally high JH titers but have downregulated JH-regulated genes in their brains [135]. In addition, ecdysteroid levels are positively correlated with ovarian development in queens [136]. The brain dopamine is positively associated with ovarian activity in reproductive workers instead of queens of *B. ignitus* [137].

Besides hormones, as a critical reproductive regulatory factor, vitellogenin is closely associated with the ovarian activation of bumblebee queens [138]. Vitellogenin has also been shown to be closely related to ovarian development in primitive eusocial sweat bees [139]. Genomes of several bumblebee species have been annotated, revealing that many genes are potentially involved in bumblebee reproduction [140,141]. Moreover, DNA methylation has been reported to be related to cooperation between workers and caste differences in bumblebees, in addition to its direct effect on caste determination, similar to its function in *A. mellifera* and other social insects [142,143,144]. Methylation-associated allele-specific expression has also been observed for two genes, ecdysone 20 monooxygenase and imaginal morphogenesis protein-late 2-like, which are essential for worker reproduction in *B. terrestris* [145]. However, the levels of these hormones and genes may be influenced by the physiological status of the queen and changes in the nesting conditions or colony populations. In the future, specific substances may be identified and incorporated into food to regulate the levels of these hormones and genes, which ultimately regulate the reproduction of the queen, especially in artificially bred bumblebee species.

## 5. Conclusions

Successful reproduction of the queen is crucial for colony development of both wild and artificially bred bumblebee species. Current studies have shown that habitat loss, extensive use of various pesticides, agricultural development, known and potentially infectious pathogens, introduction of alien honeybee and bumblebee species, and high temperatures are detrimental to bumblebee reproduction. In addition, planting flowering plants under urban conditions, mating plugs of copulated males, worker appearance at the early stage of the colony, appropriate breeding temperatures, and high titers of JH or related genes all contribute to the reproduction of bumblebees. Under natural conditions, these factors commonly positively or negatively affect colony development and reproduction. For instance, prolonged heat waves can break the flower diversity of habitats and cause the absence of food and essential nutrients, making individuals susceptible to pathogens and leading the colony to produce fewer and smaller offspring that directly threaten the next generation’s population [146]. In addition, honeybees and bumblebees compete for food resources or spread pathogens to local bumblebee species, which is dangerous to local species, especially those with small population sizes [147]. In some contexts, unique mass-flowering plants or diverse flowering landscapes can reduce the damage to foragers and larvae caused by minor infections by pathogens and small amounts of pesticides [23,58]. Hence, further studies focusing on multifactorial interaction networks are necessary to evaluate bumblebee reproduction and colony development-associated factors comprehensively. Besides this, current knowledge is mainly obtained from the artificially bred bumblebees *B. terrestris*, *B. impatiens*, *B. ignitus*, and *B. lantschouensis*, which reflect a species tendency result in some respects because the bumblebees possess high diversity and are distributed in various habitats worldwide [148,149,150,151]. Unfortunately, artificial breeding of most bumblebee species presents significant challenges that impede a comprehensive understanding of the species-specific characteristics and factors involved in reproduction and colony development. According to existing literature, optimal feed nutrition, disease prevention, and applicable queen diapause are vital factors for the successful reproduction of artificially bred bumblebees. Moreover, artificially bred bumblebees help us understand the colony itself and the relevant genes or hormones, such as mating success between males and gynes, diapause of the mated queen, and ovary development and activation, which determine the start-up of colonies and are associated with changes in the expression of many hormones or genes. These results from artificially bred bumblebees will benefit the domestication of other bumblebee species. 

Environmental and biological factors can exert synergistic or complementary effects. Understanding these factors will be invaluable for conserving wild bumblebees and breeding diverse commercial bumblebee species. However, currently known direct factors are insufficient to fully explain wild and commercial bumblebees’ reproductive success. Other factors, such as worker forage efficiency, indirectly affect colony development. More research is needed in order to further explore the biological behaviors, hormonal influences, genetic factors, and habitat changes, and to conduct an in-depth study of the known factors involved in multiphase colonies and diverse bumblebee species to identify the comprehensive impact factors. 

## Data Availability

No new data were created or analyzed in this study. Data sharing is not applicable to this article.
